# Association between patterns of biological rhythm and self-harm: evidence from the baoxing youth mental health (BYMH) cohort

**DOI:** 10.1186/s13034-023-00685-w

**Published:** 2024-01-03

**Authors:** Dan Shan, Yue Wang, Marissa Tousey-Pfarrer, Cancan Guo, Mengtong Wan, Peijie Wang, Zhihao Dai, Fenfen Ge, Jun Zhang

**Affiliations:** 1 Mental Health Center, West China Hospital, Sichuan University, Chengdu, China; 2 Department of Biobehavioral Sciences, Columbia University, New York, NY USA; 3 Center of Public Health Sciences, Faculty of Medicine, University of Iceland, Reykjavík, Iceland; 4 School of Medicine, Shanghai Jiao Tong University, Shanghai, China; 5 School of Education, Tianjin University, Tianjin, China; 6 School of Medicine, Royal College of Surgeons in Ireland, Dublin, Ireland; 7 The National Centre for Register-Based Research, Aarhus University, Aarhus, Denmark

**Keywords:** Self-harm, Biological rhythms, Suicide, Lifestyle interventions, Chinese youth

## Abstract

**Background:**

Self-harm, a severe mental health concern among children and adolescents, has varying global prevalence rates. Previous studies have suggested potential associations between specific behavioral aspects of biological rhythm and self-harm risk in these populations.

**Objective:**

Our study aimed to elucidate the relationship between biological rhythm patterns and the propensity of self-harm among Chinese children and adolescents using the Baoxing Youth Mental Health (BYMH) cohort.

**Methods:**

We included 1883 Chinese children and adolescents from the BYMH cohort. The self-report questions used to assess biological rhythm and self-harm. We applied Principal Component Analysis (PCA) to distinguish patterns of biological rhythms. Logistic regression models were conducted to estimate the associations between biological rhythm, as well as biological rhythm patterns and risk of self-harm.

**Results:**

Of the participants, 35.0% reported experiencing lifetime self-harm. PCA revealed six significantly predominant biological rhythm patterns. Elevated risks of self-harm were linked with unhealthy eating practices, daytime tiredness, and unhealthy bedtime snacking. Conversely, patterns emphasizing physical exercise, family meals for breakfast, and nutritious diet exhibited decreased self-harm propensities. These trends persisted across varied self-harm attributes, including type, recency, and frequency of self-harm.

**Conclusions:**

This study underscores the critical impact of biological rhythms on self-harm risks among Chinese youth. Targeted lifestyle interventions, focusing on improved sleep and dietary habits, could serve as potent preventive measures. Our findings lay the groundwork for future longitudinal studies to further probe these associations, fostering the creation of tailored interventions to curb self-harm and enhance mental well-being in younger populations.

**Supplementary Information:**

The online version contains supplementary material available at 10.1186/s13034-023-00685-w.

## Introduction

Self-harm, including both non-suicidal and suicidal self-harm, is a major contributor to physical harm and early death among children and adolescents, affecting approximately 14–21% of youth worldwide [21, 26, 35]. With a global lifetime prevalence of 22.0% for non-suicidal self-harm [54] and 6% for suicidal self-harm [33], understanding modifiable risk factors is crucial for primary prevention. Previous studies have highlighted potential risk factors stemming from family environments and adverse life events, such as adverse childhood experiences and assault-related incidents [34, 52]. However, there are still significant gaps in addressing potentially modifiable risks.

Biological rhythm pertains to the natural cycle of bodily functions regulated by an internal clock [42, 44]. Eating habits, physical activities, social activities, and sleep habits could be defined as the behavioral markers of this biological rhythm. Here the 'social activities' denote the habitual patterns and frequencies with which individuals partake in social interactions. Analogous to the habitual inclinations to eat, move, or sleep, people's propensity for socializing is often rhythmically patterned. Emerging evidence links biological rhythms to mood disorders in children and adolescents [4, 9], but limited research explores the relationship between biological rhythm and self-harm. Some studies have investigated the role of sleep sufficiency and physical activity in preventing self-harm [2, 6, 20, 32, 60], with a systematic review demonstrating higher non-suicidal self-harm risks among adolescents with sleep disturbances [20]. Boone et al. [6] found that lower physical activity levels correlated with increased non-suicidal self-harm frequency in youths. However, existing research primarily focuses on specific aspects of biological rhythm, leaving gaps in our comprehensive understanding of its role in preventing self-harm.

Moreover, multiple biological rhythm aspects are interconnected and may interact with each other, such as the positive association between healthy eating habits and physical activity levels [10]. Therefore, examining the associations between patterns of biological rhythm and self-harm is warranted. Additionally, while numerous studies highlighted the distinctions between non-suicidal and suicidal self-harm in terms of prevalence, risk factors, frequency, and treatment outcomes [7, 16], few studies categorize and compare these types of self-harm within the same research.

To this end, we aimed to examine the associations between patterns of biological rhythm and self-harm while controlling for known risk factors such as family environment and adverse life events [32, 39]. Furthermore, we sought to determine if these associations vary based on the type of self-harm (suicidal vs. non-suicidal), the recency of the self-harm, and the lifetime frequency of self-harm.

## Methods

### Data source and study population

The research utilized data derived from the Baoxing Youth Mental Health (BYMH) cohort. This cohort is part of an ongoing longitudinal study examining the psychosocial wellbeing of children and adolescents. With endorsement from the local Education Bureau, the cohort comprised samples from 21 primary and secondary educational institutions located in Baoxing County, Sichuan Province, China. Inclusion in the study was contingent upon obtaining informed consent from participants. Data acquisition was facilitated through traditional paper-and-pencil questionnaires, encompassing areas such as sociodemographic characteristics, daily routine and lifestyle factors, familial support, early life adversities, and both physical and psychological health conditions. Initiated in 2013, the study has successfully executed six data collection phases, as illustrated in Additional file 1: Figure S1.

For the purposes of this particular investigation, analyses were confined to a subset of 1883 participants. These individuals were involved in the sixth data collection phase, undertaken in December 2021, and provided comprehensive details regarding their biological rhythm and self-harm history, as presented in Additional file 1: Figure S2. Ethical clearance for this study was granted by the Biomedical Research Ethics Committee of West China Hospital under the reference NO. 2013[173], in conjunction with the local Education Bureau.

### Exposure

In accordance with prior research, we evaluated biological rhythms across four dimensions: eating habits, physical activities, social activities, and sleep patterns, using a specially designed questionnaire. For eating habits, we included eight questions, which assessed behaviours such as frequency of breakfast consumption, vegetable and meat intake, snacking, sharing breakfast with family, watching TV during dinner, overeating, and ordering takeout in the previous month. Physical and social activities were assessed with three and five questions respectively, with prompts like, “how frequently did you exercise post-school?” and “how often did you engage in computer games or socialize with friends?” Sleep patterns were assessed with six questions addressing issues like difficulty in initiating sleep, nighttime awakenings, early morning arousals, excessive dreaming at night, daytime dozing, and feeling sleepy during daytime. Depending on the question, responses were either based on a 5-point scale from 0 (never) to 4 (always), or a simple binary choice of 0 (no) and 1 (yes). This 22-question biological rhythms assessment showed good reliability with a Cronbach’s α value of 0.73.

### Outcome

We evaluated lifetime self-harm with the question: “Have you ever engaged in behaviours that harm your body?” Respondents could answer “yes” or “no.” Those who answered “yes” were identified as individuals with a history of self-harm. We further probed the nature of the self-harm by asking, “Did you ever attempt suicide while engaging in self-harm behaviours?” to differentiate between suicidal and non-suicidal self-harm. We also asked about the frequency of such behaviours with the question, “How many times have you engaged in self-harm in your life?” offering response options of “never,” “1–3 times,” or “more than 3 times.” Lastly, we inquired about the recency of the act with the question, “When was your most recent self-harm?” providing options of “never,” “within one month,” or “within one year.”

### Covariates

We grouped variables to see how they related to both biological rhythm patterns and self-harm. Based on prior research’s findings, we thought about age, sex, ethnicity, financial difficulties, family support, and adverse life events as possible potential covariates [14, 22, 38, 43, 50]. To check financial difficulties, we asked: “Has your family faced long-term money issues”. To know about family support, we asked: “Do you often get help or support from your family?”. Adverse life events included bullying, feeling emotional neglect, being physically or sexually abuse, domestic violence, losing family members, getting hurt in an earthquake, and problems (e.g., infection or isolation) during the COVID-19 pandemic. We further calculated the number of adverse life events experiences and classified the number as 0 and ≥ 1.

### Statistical analyses

We used percentages or means with standard deviations (SD) to present the study population’s characteristics across with or without lifetime self-harm behaviour.

### Identification of biological rhythm patterns

We identified patterns of biological rhythm using principal component analysis (PCA). Briefly, PCA offers low-dimensional information extracted from numbers of variables with high correlations, which enables the identification of distinct biological rhythm patterns of major relevance, presented as significant principal components (PCs) [61]. We retained PCs with the highest eigenvalues and cumulative explained variance over 50%. The PC scores were derived through integrating all included variables weighted via their factor loadings in the PC. Higher PC scores suggesting greater adherence to the specific patterns. For each pattern, we categorized the PC scores into low (< 1st tertile), moderate (between the 1st and 2nd tertile) and high (above the 2nd tertile) groups, and used low adherence group as the reference.

### Associations of biological rhythm and self-harm

We assessed the associations between individual items of biological rhythm and lifetime self-harm using logistic regression represented as odds ratios (OR) and their 95% confidence interval (CI). To account for multiple comparisons, we applied false discovery rate (FDR) correction in the analyses. Next, we evaluated the associations between biological rhythm patterns and suicidal and non-suicidal self-harm, recency of self-harm as well as the frequency of life-time self-harm. In all analyses, participants without lifetime self-harm were treated as the reference group. We adjusted for age (8–12 years, 13–15 years, 16–18 years), sex (boys or girls), ethnicity (Han or non-Han), financial difficulties (yes or no), family support (yes or no) and adverse life events (0 or ≥ 1).

### Missing values

We used multiple imputation (ML) to handle missing data of covariates, via predictive mean matching with m = 20 rounds of imputations. Accounting for potential bias of imputed data, we did a sensitivity analysis in which we repeated our main analysis in the complete dataset.

All analyses were performed in R 4.0 and a two-sided p value < 0.05 were defined as statistical significance.

## Results

### Characteristics of the sample

The mean (SD) age of 1883 participants were 12.3 (2.65) years and 48.2% were girls. The prevalence of lifetime self-harm was 35.0%. Compared to the reference group, participants with lifetime self-harm were more likely to be in junior high school, girls, and reported financial difficulties as well as experienced more adverse life events (Table 1).

**Table 1:** Characteristics of the study population

	Life-time self-harm behaviour		Overall (n = 1883)
	No (n = 1224)	Yes (n = 659)	
Age			
Mean (SD)	12.1 (2.76)	12.5 (2.41)	12.3 (2.65)
Median [Min, Max]	12.0 [8.00, 18.0]	13.0 [8.00, 18.0]	12.0 [8.00, 18.0]
Missing	32 (2.6%)	7 (1.1%)	39 (2.1%)
Grade			
Primary School	679 (55.5%)	297 (45.1%)	976 (51.8%)
Junior High School	332 (27.1%)	287 (43.6%)	619 (32.9%)
Senior High School	210 (17.2%)	75 (11.4%)	285 (15.1%)
Missing	3 (0.2%)	0 (0%)	3 (0.2%)
Sex			
Boys	613 (50.1%)	280 (42.5%)	893 (47.4%)
Girls	553 (45.2%)	354 (53.7%)	907 (48.2%)
Missing	58 (4.7%)	25 (3.8%)	83 (4.4%)
Ethnicity			
Han-people	930 (76.0%)	518 (78.6%)	1448 (76.9%)
Minorities	196 (16.0%)	91 (13.8%)	287 (15.2%)
Missing	98 (8.0%)	50 (7.6%)	148 (7.9%)
Financial difficulties			
No	537 (43.9%)	370 (56.1%)	907 (48.2%)
Yes	105 (8.6%)	90 (13.7%)	195 (10.4%)
Missing	582 (47.5%)	199 (30.2%)	781 (41.5%)
Family support			
No	435 (35.5%)	276 (41.9%)	711 (37.8%)
Yes	779 (63.6%)	376 (57.1%)	1155 (61.3%)
Missing	10 (0.8%)	7 (1.1%)	17 (0.9%)
Adverse life events			
No	472 (38.6%)	206 (31.3%)	678 (36.0%)
Yes	344 (28.1%)	364 (55.2%)	708 (37.6%)
Missing	408 (33.3%)	89 (13.5%)	497 (26.4%)

### Associations between biological rhythm items and lifetime self-harm

**Fig. 1:**
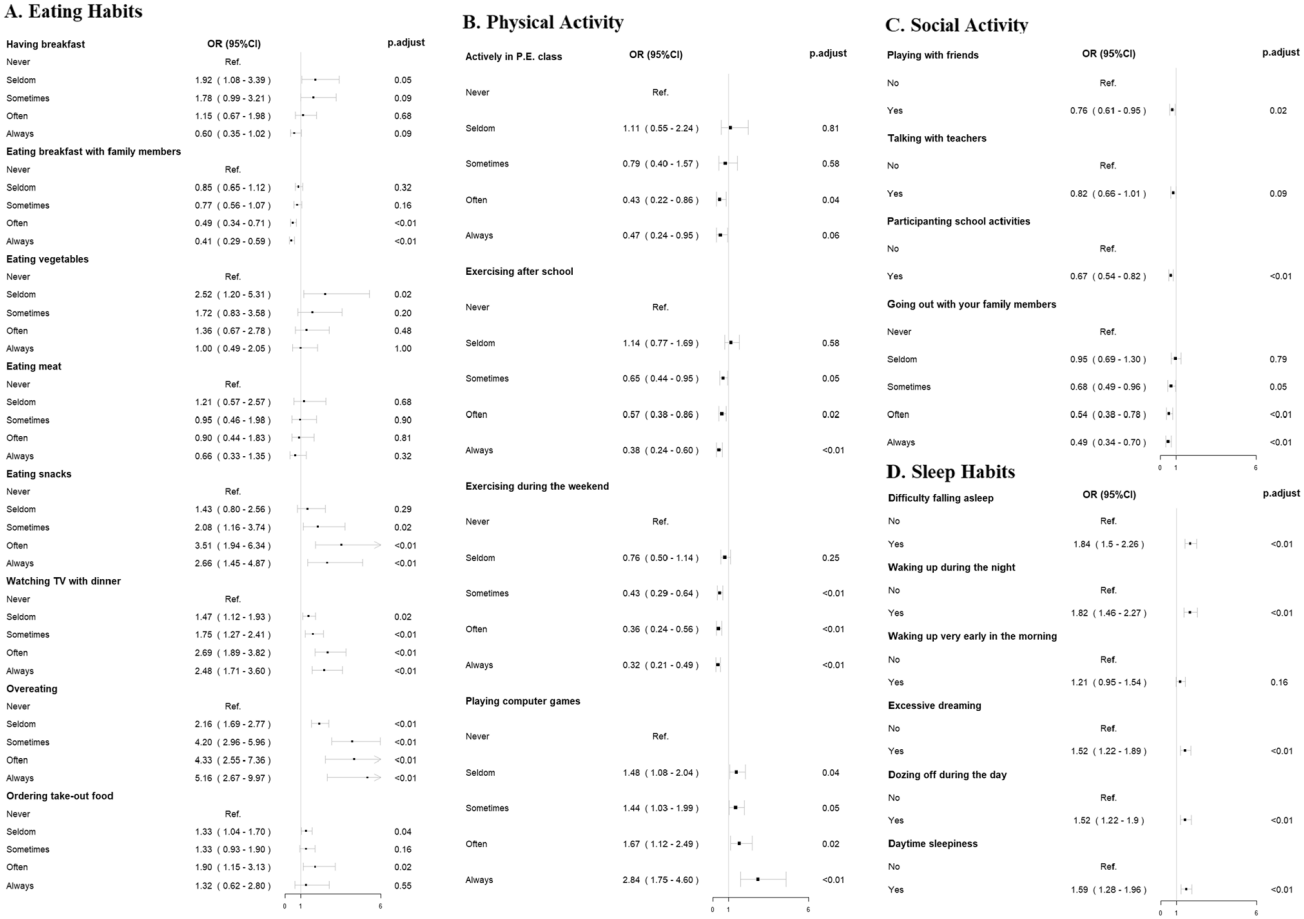
Association between single item of biological rhythm and life time self-harm behaviour

Figure 1 showed the associations between a single item of biological rhythm and lifetime self-harm. Specifically, for eating habits, eating snacks (ORs 1.43–3.51), watching TV with dinner (ORs 1.47–2.69), and overeating (ORs 2.16–5.16) was associated with a higher risk of lifetime self-harm. By contrast, a higher frequency of eating breakfast with family members was associated with a lower risk of lifetime self-harm (ORs 0.41–0.85). Compared to individuals with the lowest level (i.e., “never” or “no”), a higher level of physical and social activities was associated with a lower risk of lifetime self-harm (i.e., OR < 1), except playing computer games (ORs 1.44–2.84). For sleep habits, all six studied sleep symptoms were associated with a higher risk of lifetime self-harm (ORs 1.21 to 1.84), though the association was not statistically significant for waking up very early in the morning.

### Association of identified biological rhythm patterns and self-harm

We identified seven PCs for biological rhythm pattern, accounting for 52.2% of the total variance (Additional file 1: Figure S3). We named each PCs by using its contributing factors with the highest loading value, and labeled as “PC1 physical exercise”, “PC2 unhealthy eating practices”, “PC3 family meals for breakfast”, “PC4 nutritious diet”, “PC5 social interactions in school”, “PC6 daytime tiredness” and “PC7 unhealthy bedtime snacking”, respectively. Specifically, compared to low adherence group, high adherence level to unhealthy eating practices (OR = 2.23, 95% CI 1.71–2.92; Table 2), daytime tiredness (OR = 1.35, 95% CI 1.05–1.74), and unhealthy bedtime snacking (OR = 2.05, 95% CI 1.60–2.64) were associated with a higher risk of lifetime self-harm. Whereas high adherence to other three identified patterns were associated with reduced risk of life time self-harm, and the associations were observed for physical exercise (OR = 0.49, 95% CI 0.38–0.64), family meals for breakfast (OR = 0.52, 95% CI 0.41–0.68), nutritious diet (OR = 0.65, 95% CI 0.50–0.83). The correlation between the pattern of social interactions in school and the risk of lifetime self-harm was insignificant (OR = 0.86, 95% CI 0.67–1.12). Moreover, the associations between biological rhythm pattern and frequency of life-time self-harm behaviour were showed in a dose–response manner (Table 3).

**Table 2:** Association between patterns of biological rhythm and life time self-harm behaviour

	Lifetime self-harm behaviour, OR (95%CI)		
	Without suicidal intent	With suicidal intent	Overall
PC1 "Physical exercise"			
Low (< 1st tertile)			
Moderate (1st–2nd tertile)	0.69 (0.50–0.96)	0.90 (0.51–1.57)	0.65 (0.51–0.83)
High (≥ 2nd tertile)	0.52 (0.37–0.72)	0.70 (0.37–1.30)	0.49 (0.38–0.64)
PC2 "Unhealthy eating practices"			
Low (< 1st tertile)			
Moderate (1st–2nd tertile)	1.76 (1.26–2.47)	1.52 (0.80–2.88)	1.91 (1.47–2.48)
High (≥ 2nd tertile)	1.85 (1.30–2.64)	1.51 (0.81–2.80)	2.23 (1.71–2.92)
PC3 "Family meals for breakfast"			
Low (< 1st tertile)			
Moderate (1st–2nd tertile)	0.93 (0.67–1.28)	1.01 (0.58–1.75)	0.85 (0.67–1.09)
High (≥ 2nd tertile)	0.57 (0.41–0.80)	0.90 (0.49–1.67)	0.52 (0.41–0.68)
PC4 "Nutritious diet"			
Low (< 1st tertile)			
Moderate (1st–2nd tertile)	0.93 (0.67–1.28)	0.56 (0.31–1.02)	0.84 (0.66–1.07)
High (≥ 2nd tertile)	0.81 (0.58–1.12)	0.44 (0.24–0.82)	0.65 (0.50–0.83)
PC5 "Social interactions in school"			
Low (< 1st tertile)			
Moderate (1st–2nd tertile)	1.06 (0.77–1.46)	1.13 (0.64–1.98)	1.08 (0.85–1.38)
High (≥ 2nd tertile)	0.81 (0.58–1.14)	1.07 (0.59–1.95)	0.86 (0.67–1.12)
PC6 "Daytime tiredness"			
Low (< 1st tertile)			
Moderate (1st–2nd tertile)	0.83 (0.60–1.15)	0.79 (0.43–1.45)	0.89 (0.69–1.15)
High (≥ 2nd tertile)	1.27 (0.91–1.77)	1.13 (0.61–2.07)	1.35 (1.05–1.74)
PC7 "Unhealthy bedtime snacking"			
Low (< 1st tertile)			
Moderate (1st–2nd tertile)	0.98 (0.71–1.35)	1.89 (1.01–3.51)	1.34 (1.04–1.73)
High (≥ 2nd tertile)	1.40 (1.01–1.93)	2.67 (1.48–4.81)	2.05 (1.60–2.64)

**Table 3:** Association between patterns of biological rhythm and frequency of life time self-harm behaviour

	Frequency of lifetime self-harm behaviour, OR (95%CI)	
	1–3 times	More than 3 times
PC1 "Physical exercise"		
Low (< 1st tertile)		
Moderate (1st–2nd tertile)	0.70 (0.53–0.92)	0.56 (0.40–0.78)
High (≥ 2nd tertile)	0.54 (0.40–0.72)	0.53 (0.37–0.75)
PC2 "Unhealthy eating practices"		
Low (< 1st tertile)		
Moderate (1st–2nd tertile)	1.57 (1.18–2.08)	1.77 (1.22–2.56)
High (≥ 2nd tertile)	1.82 (1.36–2.44)	2.29 (1.57–3.34)
PC3 "Family meals for breakfast"		
Low (< 1st tertile)		
Moderate (1st–2nd tertile)	1.04 (0.79–1.36)	0.49 (0.35–0.69)
High (≥ 2nd tertile)	0.72 (0.54–0.96)	0.36 (0.25–0.51)
PC4 "Nutritious diet"		
Low (< 1st tertile)		
Moderate (1st–2nd tertile)	0.88 (0.67–1.15)	0.82 (0.58–1.15)
High (≥ 2nd tertile)	0.69 (0.53–0.92)	0.67 (0.47–0.94)
PC5 "Social interactions in school"		
Low (< 1st tertile)		
Moderate (1st–2nd tertile)	1.17 (0.89–1.54)	1.00 (0.72–1.40)
High (≥ 2nd tertile)	0.96 (0.73–1.28)	0.77 (0.54–1.11)
PC6 "Daytime tiredness"		
Low (< 1st tertile)		
Moderate (1st–2nd tertile)	0.81 (0.61–1.07)	0.91 (0.64–1.31)
High (≥ 2nd tertile)	1.30 (0.98–1.72)	1.46 (1.02–2.09)
PC7 "Unhealthy bedtime snacking"		
Low (< 1st tertile)		
Moderate (1st–2nd tertile)	1.34 (1.01–1.76)	1.53 (1.05–2.24)
High (≥ 2nd tertile)	1.72 (1.30–2.28)	2.59 (1.81–3.72)

Similar results were observed when stratified by lifetime suicidal and non-suicidal self-harm (Table 2), and recency of self-harm (Table 4). Still, the associations partly did not reach significance anymore when stratifying by lifetime suicidal versus non-suicidal self-harm. In addition, we observed largely comparable results patterns when restricted analysis in the complete cases (Additional file 1: Table S1–S3).

**Table 4:** Association between patterns of biological rhythm and recency of self-harm behaviour

	Recency of self-harm behaviour, OR (95%CI)	
	Within 1 month	Within 1 year
PC1 "Physical exercise"		
Low (< 1st tertile)		
Moderate (1st–2nd tertile)	0.67 (0.49–0.92)	0.62 (0.48–0.81)
High (≥ 2nd tertile)	0.51 (0.36–0.72)	0.54 (0.41–0.72)
PC2 "Unhealthy eating practices"		
Low (< 1st tertile)		
Moderate (1st–2nd tertile)	1.36 (0.96–1.93)	1.61 (1.21–2.15)
High (≥ 2nd tertile)	1.69 (1.19–2.39)	2.06 (1.53–2.75)
PC3 "Family meals for breakfast"		
Low (< 1st tertile)		
Moderate (1st–2nd tertile)	0.76 (0.55–1.05)	0.76 (0.58–0.99)
High (≥ 2nd tertile)	0.57 (0.40–0.80)	0.48 (0.36–0.64)
PC4 "Nutritious diet"		
Low (< 1st tertile)		
Moderate (1st–2nd tertile)	0.87 (0.63–1.20)	0.98 (0.75–1.27)
High (≥ 2nd tertile)	0.73 (0.52–1.02)	0.72 (0.55–0.95)
PC5 "Social interactions in school"		
Low (< 1st tertile)		
Moderate (1st–2nd tertile)	0.91 (0.66–1.25)	1.13 (0.86–1.48)
High (≥ 2nd tertile)	0.87 (0.62–1.22)	1.04 (0.78–1.38)
PC6 "Daytime tiredness"		
Low (< 1st tertile)		
Moderate (1st–2nd tertile)	0.89 (0.63–1.25)	0.72 (0.54–0.95)
High (≥ 2nd tertile)	1.19 (0.85–1.67)	1.10 (0.84–1.46)
PC7 "Unhealthy bedtime snacking"		
Low (< 1st tertile)		
Moderate (1st–2nd tertile)	1.30 (0.92–1.84)	1.40 (1.05–1.87)
High (≥ 2nd tertile)	1.54 (1.10–2.15)	2.21 (1.67–2.92)

## Discussion

In the BYMH cohort, our study indicated that 35.0% of Chinese children and adolescents reported lifetime self-harm, with biological rhythms playing a significant role in influencing this risk. Using PCA based on single items of biological rhythm, we identified six unique patterns that appeared to influence the risk of self-harm statistically significantly. Our findings suggest that individuals with high adherence to unhealthy eating practices, daytime tiredness, and unhealthy bedtime snacking were at greater risk for lifetime self-harm. In contrast, those with high adherence to physical exercise, family meals for breakfast, and nutritious diet showed a reduced risk of lifetime self-harm. These associations remained consistent across various characteristics of self-harm, including lifetime suicidal and non-suicidal self-harm, stratifying by recency of self-harm behaviours, and the frequency of life-time self-harm behaviours, emphasizing the potential of biorhythm interventions in primary prevention strategies for self-harm among children and adolescents.

A meta-analysis included 136 studies found that lifetime self-harm ranges from 21% to 25% among adolescents (10 to 19 years old) in China, our result was slightly higher than it [26]. The variation may be explained by methodological divergences, as well as geographical influences. Of note, most prior studies have focused on the nutritional components of an individual's diet among adults, few have explored specific eating patterns, such as family meals for breakfast and unhealthy bedtime snacking, in relation to self-harm. Overall, our findings extended the previous findings. Specifically, our study is consistent with previous literature directly or indirectly, suggesting that unhealthy eating practices, daytime tiredness, and unhealthy bedtime snacks significantly increased the risk of lifetime self-harm [20, 28, 49].

Chinese adolescents with a preference for night snacking were found more likely to have mental health problems [25]. Unhealthy eating patterns, such as snacking instead of meals, were associated with conduct/oppositional disorders, contributing to increased risks of self-harm [1, 30, 36, 48]. Daytime tiredness indicates insufficient sleep quality, a known risk factor for self-harming behaviours [29, 31, 32]. This sleep disturbance is associated with increased irritability, impulsivity, and impaired decision-making, making it difficult to cope with stressors and regulate emotions [11, 19]. As a result, the likelihood of engaging in self-harm behaviours is raised [20]. Our study extended previous findings by identifying night snacking, snacking instead of meals, and excessive daytime sleepiness (an indicator of poor sleep quality) as factors increasing the risk of self-harm in children and adolescents. Indeed, randomized controlled trials (RCTs) have found that promoting sleep quality can effectively intervene in self-harm risk factors among adults [45]. Thus, our study provides additional evidence and targets for further exploration of relevant interventions to improve sleep quality in children and adolescents at risk for self-harm.

On the other hand, adherence to regular physical exercise, family meals for breakfast, and a nutritious diet can reduce the risk of lifetime self-harm. Latina et al. [24] found that adolescents who engaged in organized sports activities were more likely to have increased self-esteem one year later and a decreased likelihood of self-harming behaviours. Berg et al. [5] found that poor diets were associated with increased risks of deliberate self-harm episodes. Agathão et al. [3] found that a lack of family meals was associated with a higher frequency of mental disorders. Park et al. [37] found that breakfast consumption played a crucial role in emotional stability. Skipping it was notably linked to a increased risk of suicidal tendencies in adolescents, as evidenced by an increase in suicide attempts. Moreover, we observed an inverse dose–response effect across these three patterns, suggesting that as their frequency increased, the risk of self-harm decreased. However, this relationship may not be linear, and there may be a threshold or optimal level of these behaviours that provides the greatest benefit in terms of reducing the risk of self-harm. Future research should explore this possibility further.

Similar associations were found across various characteristics of self-harm, including self-harm with or without suicidal intent, recency of self-harm behaviours, and frequency of lifetime self-harm behaviours. These findings suggest that the same risk and protective factors, including unhealthy eating practices, daytime tiredness, unhealthy bedtime snacks, physical exercise, family meals for breakfast, and a nutritious diet have a comparable influence on these different characteristics of self-harm. Several recent studies have investigated the association between disordered biological rhythm patterns and emotional symptoms, and pointed out that disordered biological rhythm patterns, such as problematic sleep and eating habits rhythms, could directly or indirectly induce depressive-like symptoms [27, 59], which are highly related to self-harm behaviours. However, these studies have shared similar methodological flaws. Biological rhythm typically reflects an individual's long-standing, lifestyle-related behavioral patterns [40]. In these studies, researchers only evaluated the participants' depression or anxiety symptoms as outcomes in the past 2 weeks via psychiatric questionnaires [27, 59]. Thus, although the researchers adjusted for demographic confounding factors, the lack of evaluation on the long-term emotional symptoms of these participants leads to contingency bias, resulting in decreasing the validity of such associations. In contrast, our study examined two temporal characteristics (i.e., recency of self-harm) of participants' self-harm behaviours and suggested consistent findings, thereby enhancing the validity and reliability of our results. Furthermore, although the Biological Rhythms Assessment in Neuropsychiatry (BRAIN) and self-rating of biological rhythm disorder for adolescents (SBRDA) utilized in prior studies are effective tools in evaluating neuropsychiatric conditions [55, 59], our study utilized a PCA model that provided a more objective way to identify certain biological rhythm patterns associated with self-harm behaviours by integrating diverse single items, preventing the omission of potentially essential items or patterns associated with self-harm. Additionally, we separately evaluated self-harm with and without suicidal intent and revealed consistent findings, enhancing the understanding of biological rhythm patterns in both conditions.

## Strengths and limitations

One key strength of our study is the utilization of principal component analysis (PCA) to identify biological rhythm patterns associated with self-harm, providing a more comprehensive understanding of their effects on self-harm risk. This method allowed us to examine the complex interactions between various biological rhythm components, which is a departure from previous research that focused on individual items or total scores of questionnaires [27, 55, 59]. Another strength is our consideration of potential confounding factors, such as traumatic life events (e.g., post-earthquake mental complications and COVID-19-related experiences), which may have exacerbated mental health challenges and disrupted biological rhythms among children and adolescents [46, 47, 56]. In our analyses, we carefully controlled for the specific effects of these adverse events, including earthquakes and the COVID-19 pandemic, to the greatest extent possible. Furthermore, our detailed questionnaires allowed us to explore the different characteristics of self-harm, including type of self-harm (suicidal vs. non-suicidal), the recency of the self-harm, and the lifetime frequency of self-harm.

Nonetheless, some limitations should be noted. First, our self-constructed measurement of biological rhythm may be prone to information bias and has not undergone validation. Second, the cross-sectional design of our study precludes causal inferences, necessitating future longitudinal research to examine the temporal relationship between biological rhythm patterns and self-harm. Third, our findings are based on a sample from a single region in China, which may limit the generalizability of the results due to the specific age range and sociodemographic characteristics of the participants. Fourth, self-reported data may be subjected to recall and reporting bias, which could potentially affect the accuracy of our findings. Lastly, although we controlled for many potential confounders, residual confounding may still exist due to unmeasured factors.

Considering previous findings, guidelines as well as our findings, we propose six practical evidence-based recommendations to address modifiable self-harm risk factors for children and adolescents:

**Table:** 

1. Encourage mindful eating practices, undistracted mealtimes, awareness of satiety cues, and promote nutritious home-cooked meals over take-out food consumption to foster healthier eating habits in children and adolescents [53]
2. Reduce daytime sleepiness through regular moderate-intensity exercise, such as brisk walking or swimming, for at least 30 min on most days [51]
3. Facilitate sleep onset by practicing good sleep hygiene, including a consistent bedtime, engaging in calming pre-sleep activities, and minimizing electronic device usage before sleep [17]
4. Incorporate a diverse range of age-appropriate aerobic, strength, and flexibility exercises into a weekly routine and school-based P.E. classes to improve overall physical and mental health [23]
5. Ensure daily consumption of a nutrient-dense breakfast, comprising whole grains, fruits, and protein, which has been linked to enhanced cognitive function, mood regulation, and reduced impulsivity [37, 41]
6. Promote a varied diet that includes fruits, vegetables, whole grains, lean proteins, and healthy fats for children and adolescents to optimize nutrient intake and advocate avoiding snacking as much as possible [18]

Overall, our study represents a novel approach in investigating the association between biological rhythm patterns and self-harm among Chinese children and adolescents, by incorporating and synthesizing components involved in the same type of biological rhythm pattern. The innovative methodology utilized in this study offers valuable insights into the relationship between various risk factors and self-harm. However, additional longitudinal research is needed to establish the temporal relationship between biological rhythm patterns and self-harm and to explore the complex interplay among these patterns in diverse populations. Such research can help inform the development of effective interventions to prevent self-harm and promote mental health and wellbeing in children and adolescents.

## Conclusion

Our study within the BYMH cohort shed light on the profound relationship between biological rhythms and the risk of self-harm in Chinese children and adolescents. A significant 35.0% reported self-harm behaviour in a lifetime, with detrimental eating and sleeping habits amplifying this risk, while positive behaviours like regular exercise and nutritious eating patterns mitigated it. While our findings echoed and expanded upon past research, there were inherent limitations, emphasizing the need for future longitudinal studies. Ultimately, understanding these biological rhythms is crucial for crafting effective interventions to bolster mental health and prevent self-harm in youth.

### Supplementary Information

**Additional file 1: Figure S1** Timeline of data collection. **Figure S2** Flowchart of the study population. **Figure S3** Patterns of Biological Rhythm. **Table S1 **Association between patterns of biological rhythm and life time self-harm behaviour, complete dataset analysis. **Table S2 **Association between patterns of biological rhythm and recency of self-harm behaviour, complete dataset analysis. **Table S3 **Association between patterns of biological rhythm and frequency of life-time self-harm behaviour, complete dataset analysis.

## Data Availability

The original contributions presented in the study are included in the article/Supplementary material, further inquiries can be directed to the corresponding authors.
