# Supplementary material for: Association between patterns of biological rhythm and self-harm: evidence from the baoxing youth mental health (BYMH) cohort
Source: Child Adolesc Psychiatry Ment Health. 2024 Jan 3;18:3. doi: 10.1186/s13034-023-00685-w (PMC10765742; doi:10.1186/s13034-023-00685-w)
Supplement: Supplementary file 1 — Additional file 1: Figure S1 Timeline of data collection. Figure S2 Flowchart of the study population. Figure S3 Patterns of Biological Rhythm. Table S1 Association between patterns of biological rhythm and life time self-harm behaviour, complete dataset analysis. Table S2 Association between patterns of biological rhythm and recency of self-harm behaviour, complete dataset analysis. Table S3 Association between patterns of biological rhythm and frequency of life-time self-harm behaviour, complete dataset analysis. [file 13034_2023_685_MOESM1_ESM.docx]

**sFigure 1** Timeline of data collection


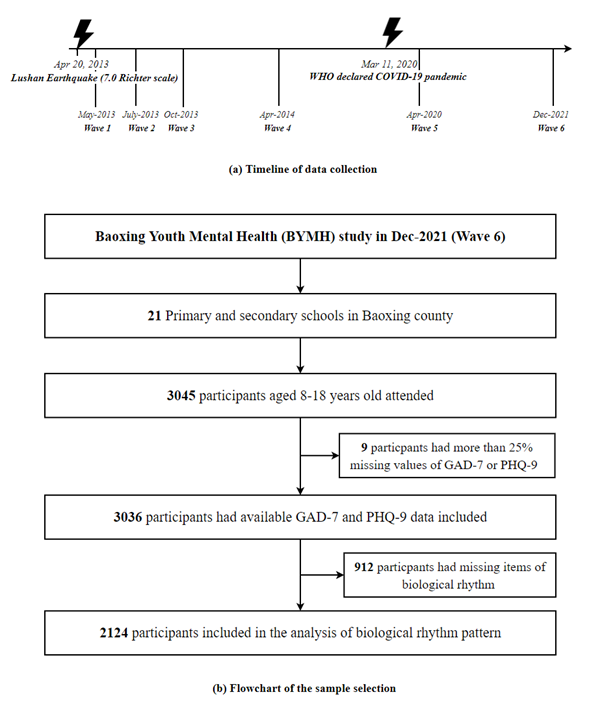


**sFigure 2** Flowchart of the study population


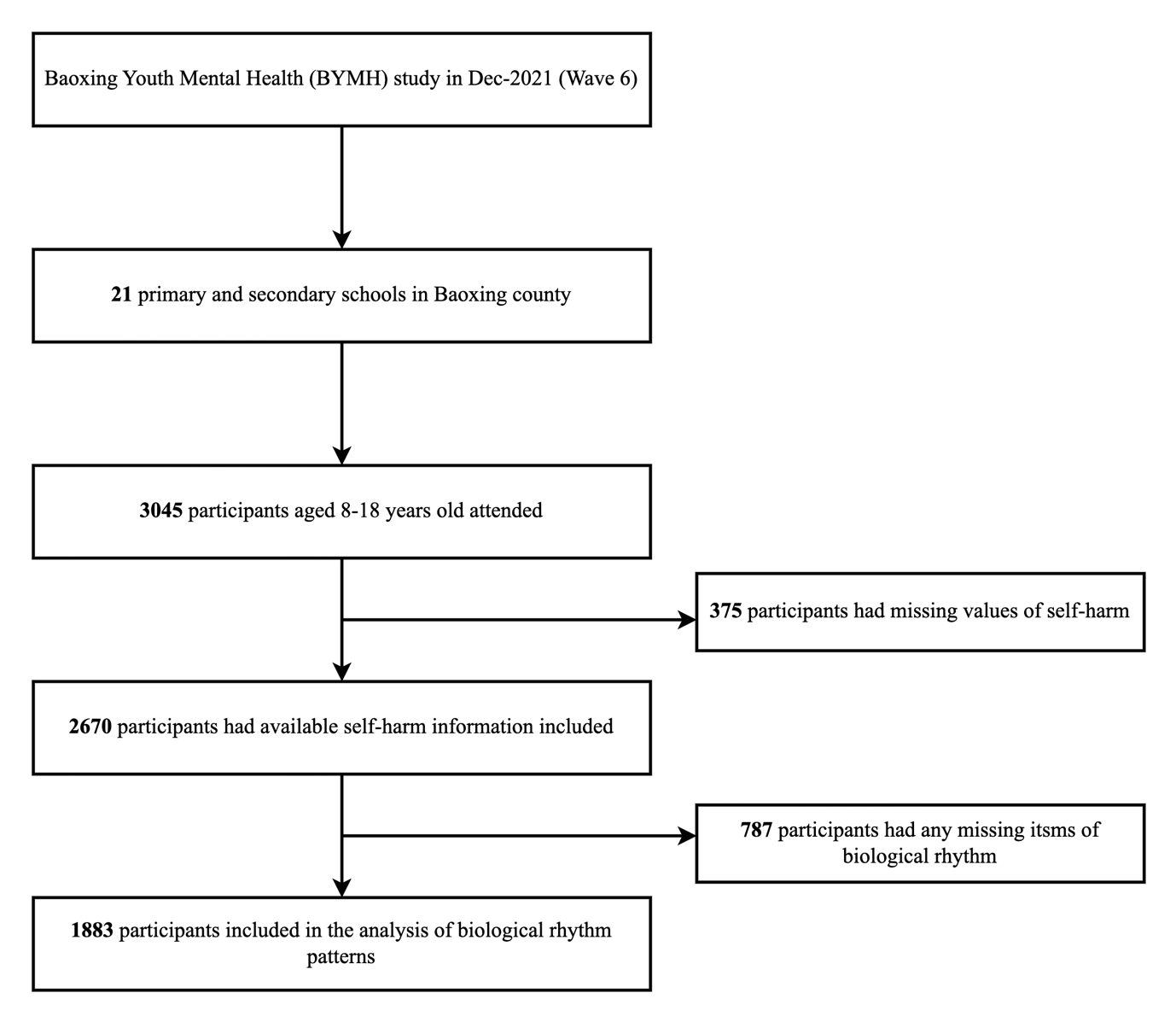


**sFigure 3** Patterns of Biological Rhythm
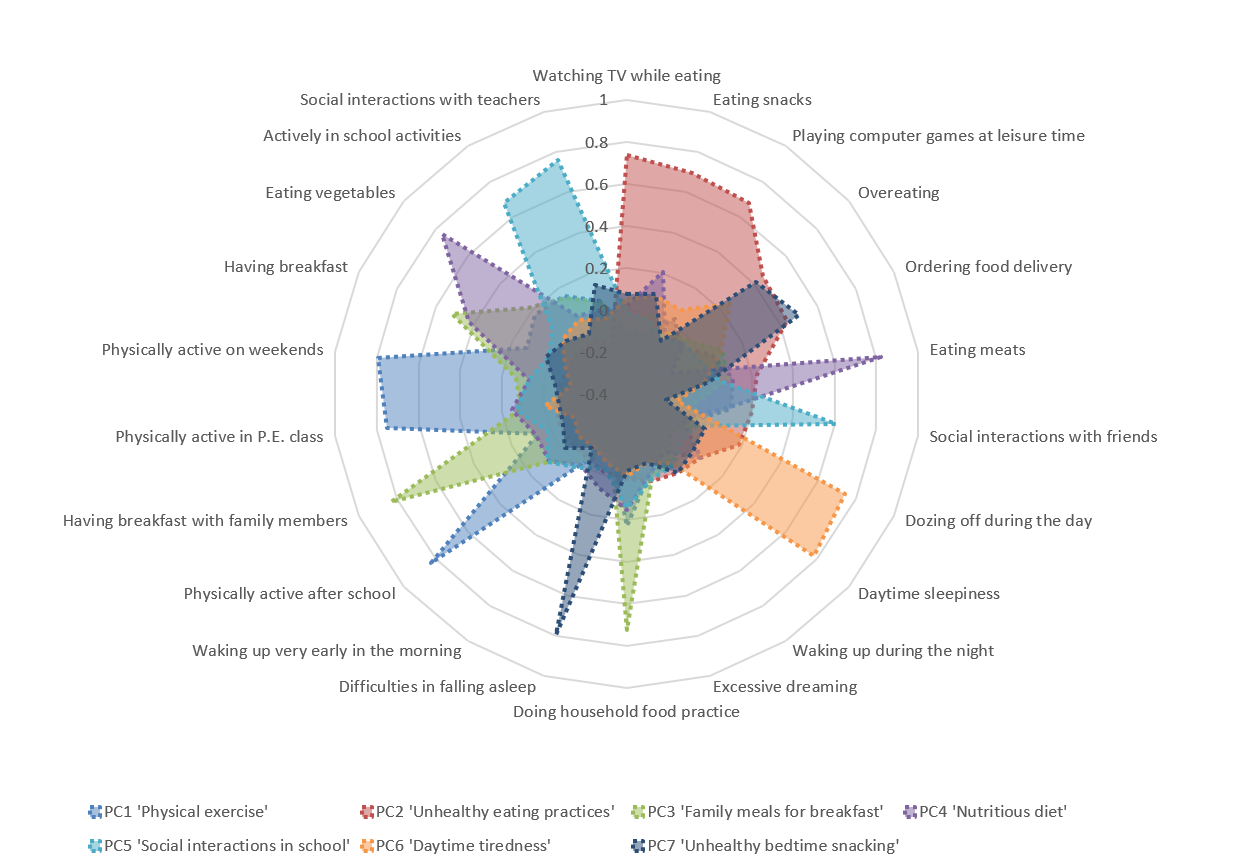


**sTable 1** Association between patterns of biological rhythm and life time self-harm behavior, complete dataset analysis

|  | **Lifetime self-harm** | | |
| --- | --- | --- | --- |
|  | **Without Suicide Idea** | **With Suicide Idea** | **Overall** |
| **PC1 "Physical exercise"** |  |  |  |
| Low (< 1st tertile) |  |  |  |
| Moderate (1st - 2nd tertile) | 0.84 (0.54 - 1.30) | 1.10 (0.53 - 2.29) | 0.80 (0.58 - 1.11) |
| High (≥2nd tertile) | 0.70 (0.45 - 1.09) | 0.82 (0.37 - 1.84) | 0.62 (0.44 - 0.88) |
| **PC2 "Unhealthy eating practices"** | |  |  |
| Low (< 1st tertile) |  |  |  |
| Moderate (1st - 2nd tertile) | 1.85 (1.20 - 2.86) | 1.22 (0.53 - 2.77) | 1.90 (1.35 - 2.69) |
| High (≥2nd tertile) | 1.56 (0.97 - 2.50) | 1.27 (0.56 - 2.78) | 2.04 (1.43 - 2.92) |
| **PC3 "Family meals for breakfast"** | |  |  |
| Low (< 1st tertile) |  |  |  |
| Moderate (1st - 2nd tertile) | 0.84 (0.55 - 1.29) | 0.9 (0.44 - 1.83) | 0.78 (0.57 - 1.08) |
| High (≥2nd tertile) | 0.67 (0.43 - 1.04) | 0.8 (0.36 - 1.79) | 0.63 (0.45 - 0.88) |
| **PC4 "Nutritious diet"** |  |  |  |
| Low (< 1st tertile) |  |  |  |
| Moderate (1st - 2nd tertile) | 0.83 (0.54 - 1.27) | 0.30 (0.11 - 0.72) | 0.73 (0.53 - 1.01) |
| High (≥2nd tertile) | 0.91 (0.59 - 1.40) | 0.16 (0.06 - 0.38) | 0.67 (0.48 - 0.94) |
| **PC5 "Social interactions in school"** |  |  |  |
| Low (< 1st tertile) |  |  |  |
| Moderate (1st - 2nd tertile) | 1.14 (0.74 - 1.74) | 2.35 (1.14 - 5.06) | 1.24 (0.89 - 1.72) |
| High (≥2nd tertile) | 0.72 (0.46 - 1.13) | 1.39 (0.66 - 2.98) | 0.82 (0.58 - 1.16) |
| **PC6 "Daytime tiredness"** | |  |  |
| Low (< 1st tertile) |  |  |  |
| Moderate (1st - 2nd tertile) | 0.89 (0.58 - 1.37) | 0.87 (0.39 - 1.91) | 0.98 (0.70 - 1.38) |
| High (≥2nd tertile) | 1.19 (0.76 - 1.85) | 1.22 (0.55 - 2.64) | 1.43 (1.02 - 2.01) |
| **PC7 "Unhealthy bedtime snacking"** |  |  |  |
| Low (< 1st tertile) |  |  |  |
| Moderate (1st - 2nd tertile) | 0.87 (0.57 - 1.32) | 2.17 (0.98 - 4.88) | 1.23 (0.88 - 1.73) |
| High (≥2nd tertile) | 1.31 (0.84 - 2.03) | 3.00 (1.42 - 6.40) | 2.06 (1.46 - 2.90) |

**sTable 2** Association between patterns of biological rhythm and recency of self-harm behavior, complete dataset analysis

|  | **OR (95%CI)** | |
| --- | --- | --- |
|  | **Within 1 month** | **Within 1 year** |
| **PC1 "Physical exercise"** |  |  |
| Low (< 1st tertile) |  |  |
| Moderate (1st - 2nd tertile) | 0.78 (0.52 - 1.17) | 0.75 (0.53 - 1.06) |
| High (≥2nd tertile) | 0.58 (0.37 - 0.91) | 0.54 (0.37 - 0.79) |
| **PC2 "Unhealthy eating practices"** |  |  |
| Low (< 1st tertile) |  |  |
| Moderate (1st - 2nd tertile) | 1.12 (0.72 - 1.74) | 1.52 (1.04 - 2.23) |
| High (≥2nd tertile) | 1.51 (0.97 - 2.36) | 2.17 (1.47 - 3.22) |
| **PC3 "Family meals for breakfast"** |  |  |
| Low (< 1st tertile) |  |  |
| Moderate (1st - 2nd tertile) | 0.74 (0.49 - 1.11) | 0.71 (0.50 - 1.01) |
| High (≥2nd tertile) | 0.70 (0.45 - 1.08) | 0.58 (0.40 - 0.84) |
| **PC4 "Nutritious diet"** |  |  |
| Low (< 1st tertile) |  |  |
| Moderate (1st - 2nd tertile) | 0.65 (0.43 - 0.99) | 0.97 (0.68 - 1.38) |
| High (≥2nd tertile) | 0.62 (0.40 - 0.94) | 0.73 (0.50 - 1.06) |
| **PC5 "Social interactions in school"** |  |  |
| Low (< 1st tertile) |  |  |
| Moderate (1st - 2nd tertile) | 0.78 (0.51 - 1.18) | 1.30 (0.91 - 1.86) |
| High (≥2nd tertile) | 0.79 (0.51 - 1.21) | 1.06 (0.72 - 1.55) |
| **PC6 "Daytime tiredness"** |  |  |
| Low (< 1st tertile) |  |  |
| Moderate (1st - 2nd tertile) | 1.10 (0.70 - 1.72) | 0.91 (0.62 - 1.33) |
| High (≥2nd tertile) | 1.46 (0.94 - 2.27) | 1.26 (0.87 - 1.84) |
| **PC7 "Unhealthy bedtime snacking"** |  |  |
| Low (< 1st tertile) |  |  |
| Moderate (1st - 2nd tertile) | 1.17 (0.75 - 1.82) | 1.16 (0.79 - 1.70) |
| High (≥2nd tertile) | 1.38 (0.90 - 2.14) | 2.17 (1.50 - 3.16) |

**sTable 3** Association between patterns of biological rhythm and frequency of life-time self-harm behavior, complete dataset analysis

|  | **Without self-harm behaviour as the reference, OR (95%CI)** | |
| --- | --- | --- |
|  | **1-3 times** | **>3 times** |
| **PC1 "Physical exercise"** | |  |
| Low (< 1st tertile) |  |  |
| Moderate (1st - 2nd tertile) | 1.01 (0.70 - 1.46) | 0.71 (0.46 - 1.10) |
| High (≥2nd tertile) | 0.77 (0.52 - 1.14) | 0.55 (0.35 - 0.89) |
| **PC2 "Unhealthy eating practices"** | |  |
| Low (< 1st tertile) |  |  |
| Moderate (1st - 2nd tertile) | 1.57 (1.08 - 2.29) | 1.71 (1.07 - 2.75) |
| High (≥2nd tertile) | 1.68 (1.13 - 2.50) | 2.08 (1.28 - 3.36) |
| **PC3 "Family meals for breakfast"** | |  |
| Low (< 1st tertile) |  |  |
| Moderate (1st - 2nd tertile) | 1.00 (0.69 - 1.45) | 0.45 (0.29 - 0.70) |
| High (≥2nd tertile) | 0.95 (0.65 - 1.40) | 0.42 (0.26 - 0.67) |
| **PC4 "Nutritious diet"** | |  |
| Low (< 1st tertile) |  |  |
| Moderate (1st - 2nd tertile) | 0.79 (0.55 - 1.14) | 0.68 (0.43 - 1.05) |
| High (≥2nd tertile) | 0.68 (0.47 - 0.99) | 0.71 (0.45 - 1.10) |
| **PC5 "Social interactions in school"** | |  |
| Low (< 1st tertile) |  |  |
| Moderate (1st - 2nd tertile) | 1.29 (0.89 - 1.88) | 1.08 (0.70 - 1.66) |
| High (≥2nd tertile) | 1.04 (0.71 - 1.52) | 0.66 (0.41 - 1.07) |
| **PC6 "Daytime tiredness"** | |  |
| Low (< 1st tertile) |  |  |
| Moderate (1st - 2nd tertile) | 0.82 (0.56 - 1.20) | 0.91 (0.57 - 1.45) |
| High (≥2nd tertile) | 1.44 (0.98 - 2.10) | 1.32 (0.83 - 2.10) |
| **PC7 "Unhealthy bedtime snacking"** | |  |
| Low (< 1st tertile) |  |  |
| Moderate (1st - 2nd tertile) | 1.16 (0.80 - 1.68) | 1.30 (0.80 - 2.11) |
| High (≥2nd tertile) | 1.56 (1.07 - 2.28) | 2.38 (1.49 - 3.80) |
